# Is there a need for creators of imaginary authors to face legal consequences?

**DOI:** 10.3325/cmj.2020.61.561

**Published:** 2020-12

**Authors:** Jaime A. Teixeira da Silva

**Affiliations:** Independent scholar, Kagawa-ken, Japan *jaimetex@yahoo.com*

Academic publishing is increasingly threatened by unscholarly forces, including false and/or predatory entities. One serious false element are individuals that masquerade as fictitious authors by creating fictitious personalities, emails, and affiliations, sometimes misappropriating the entities of real scholars. One objective of fictitious papers is to target “predatory” (ie, illegitimate and unscholarly) as well as legitimate (ie, indexed and metricized) journals or publishers, possibly to expose flawed peer review. Some fictitious papers are retracted, but retractions are insufficient since the real individuals who created fictitious authors and papers suffer no ethical or legal consequences.

To exemplify this threat, academics should pay attention to the retraction of the “Bo Liu” et al study in Elsevier’s *The International Journal of Biochemistry & Cell Biology* (*IJB&CB*). This is not a sting operation or hoax paper (1). It is a case of unprecedented deceit that threatens academic publishing, and is a new form of “predatory publishing” in the coronavirus disease 2019 (COVID-19) era because it preys upon the ingenuity of open access and subscription predatory and indexed, peer-reviewed journals (2). Predatory publishing is a waste of resources (3), a destabilizing force that disrupts “the control of scholarly communication by for-profit multi-national corporations” (p. 176) (4), or consumer fraud (5). The latter was substantiated by a high-profile US Federal Trade Commission vs OMICS International case (6,7). Do the actions underlying the “Bo Liu” et al study fall under the False Statements Act? Are legal consequences such as criminal penalties justified in academia, which appears to be increasingly “militarized” (8)? Even though Hickman et al (9) suggested criminal liability for metrics manipulation or for coercive citation tactics by authors or editors, the criminalization of research misconduct faces multiple challenges and limitations (10). LexisNexis, a legal firm in RELX Group, Elsevier’s parent organization, appears to have sufficient forensic, legal, and financial resources to conduct a more in-depth investigation.

If cases like this proliferate, the ability to discern valid from invalid authors, institutions, and academic papers, will fade. The most prominent issues underlying the retracted “Bo Liu” et al paper are false and/or misappropriated authorship and institutional affiliations. Peer review can sometimes fail to identify such false elements (11). A retraction, the evident outcome, is therefore an absolutely insufficient remediation. How many papers are there with false authors, either in “valid” (ie, indexed on a major database like PubMed, Scopus, or Web of Science, or with a journal-based metric such as a journal impact factor or CiteScore), or in predatory venues? One underlying bibliometric concern is the attribution of citations to false authors and to journals that publish papers written by them (12). Rivera (13) refers to the phenomenon of false authorship as “evil,” but the suggestion that “the commitment of Publons to strengthen this fundamental process and ultimately ensure the quality and integrity of the published articles is laudable” is disingenuous since papers with false elements, such as the “Bo Liu” et al paper, are “indexed” at Publons, suggesting that this reviewer reward platform may be part of the problem in academia, serving as a catalyst, and not just a solution (14).

Researcher networks like Mendeley, ResearchGate, or ORCID, an author disambiguation tool, may become untrustworthy if they are increasingly populated by false identities. The indexing of false or predatory entities may affect the accuracy and reputation of major databases such as PubMed, Scopus, or Web of Science (15), or even the Retraction Watch retraction database. For example, a September 21, 2020 search on the latter database for “Liu, Bo” revealed that the false “Bo Liu” is in one of 26 entries (16 entries are false positives) involving an author named “Bo Liu.” Retraction Watch also covered this case.

Screening and detection by peers and editors was unable to prevent this paper with false elements from being published, so editors should be held accountable to some extent (16), although editorial oversight resulting from deception by false authors should be forgiven without attaching a stigma to it (17). The relative opacity of the retraction notice is another problem (18).

A bevy of ethical infractions characterize the “Bo Liu” et al case: false authorship, concocted emails, misappropriated institutions, identity theft, and reputational hijacking. All of these feed into the notion that “fake” elements are increasing in academic publishing (19). Was a paper-mill involved (20,21)? What is the nationality of the “Bo Liu” creators (8 out of the 10 listed “authors” have Chinese names and 4 out of the 7 listed affiliations are in China)? Was their intent to discredit Chinese academia, ie, a novel form of racism (22)? Or was the paper an act of anti-status quo (in this case Elsevier) rebellion?

*IJB&CB* is by many academics’ standards a “respectable” journal: it has claimed peer review and a large and apparently reputable editor board, but editors’ conflicts of interests are missing (23); the publisher (Elsevier) is famous and one of the largest academic publishers; it is indexed in Scopus and on about a dozen other indexes; it has a 2019 CiteScore of 3.29 and a 2019 Clarivate Analytics journal impact factor of 3.144; a rich history, and 125 volumes to boast. *IJB&CB* has ample characteristics of what academics might consider to be a non-predatory, white-listed, valid academic and scholarly journal (24).

There are four retractions in *IJB&CB*, including the one by “Bo Liu” et al, so this is not a black swan event because *IJB&CB* has prior experience and should have been prepared, but was not. The “Bo Liu” et al paper has, according to Google Scholar, been cited once (25), and access to a pirated copy at Sci-Hub might allow academics to cite it (26). Citation of the retracted *IJB&CB* paper offers misleading and unfair “recognition” to its “authors” and may represent a tactical victory to the entity(ies) that created “Bo Liu” and this false paper.

The author disambiguation tool, ORCID, had (September 21, 2020) over 340 “Bo Liu” or “Liu Bo” registered, most of which are “single-use” accounts with no identifying information that could shed light on the real identity of the lead author or confirm if any of them is the “Bo Liu” of the now retracted *IJB&CB* paper. Since the ORCID mission failed in this case because this tool could not disambiguate real from actual or potentially false authors, ORCID may also thus begin to represent an academic threat.

Ethics groups and organizations such as Committee on Publication Ethics, International Committee of Medical Journal Editors, Open Access Scholarly Publishers Association, and Directory of Open Access Journals, which collectively represent thousands of publishers and journals, need to take action and assume a clear and resolute stance regarding the creators of fake elements such as false authors or emails, or individuals who misappropriate institutions because these actions cause reputational and financial damage to valid authors, journals, and publishers. In addition to labeling such actions as profoundly unethical, instituting life-time bans in affected journals and publishers, stronger detection and prevention methods need to be implemented (27).
